# Efficacy of Multimodal Rehabilitation Protocol with High-Force Machine Spinal Decompression Therapy in Chronic Low Back Pain with Sciatica Due to Lumbar Disc Herniation: A Pre–Post Observational Study

**DOI:** 10.3390/healthcare14101294

**Published:** 2026-05-10

**Authors:** Bernard B. N. Nado, Snježana Schuster

**Affiliations:** 1 Department of Rehabilitation and Physical Therapy, Polyclinics Nado Centar, 10000 Zagreb, Croatia; 2 Department of Physiotherapy, Alma Mater Europaea, 2000 Maribor, Slovenia; snjezana.schuster@zvu.hr; 3 Faculty of Kinesiology, University of Zagreb, 10000 Zagreb, Croatia; 4 Department of Physiotherapy, University of Applied Health Sciences Zagreb, 10000 Zagreb, Croatia

**Keywords:** lumbar disc herniation, sciatica, spinal decompression, traction, chronic low back pain

## Abstract

Background: Chronic low back pain (CLBP) with sciatica caused by lumbar disc herniation is a common and disabling condition. Combined therapeutic protocols that include high force machine spinal decompression therapy (SDT), infrared therapy, and interferential therapy are increasingly used in clinical practice, although evidence in chronic populations remains limited. Because this study did not include a control group, only observed pre–post changes can be reported. This study primarily aimed to assess observed changes in pain intensity following a multimodal therapy protocol in adults with chronic lumbar radiculopathy. Methods: A pre–post observational study was conducted in 234 adults with chronic lumbar radiculopathy lasting ≥12 weeks and MRI confirmed disc herniation at L4–L5 and/or L5–S1. Participants completed ten treatment sessions delivered twice weekly over five weeks. Each session included infrared therapy, high force SDT, and interferential therapy. Pain intensity (VAS 0–10) was measured before the first and before the tenth session. Results: Pain intensity decreased significantly after treatment (Wilcoxon W = 18,830, *p* < 0.001), with a mean reduction of 2.5 points, exceeding the minimal clinically important change threshold, and with a very large effect size (rank biserial correlation = 0.991). No significant gender differences were observed. Baseline pain (β = 0.312, *p* < 0.001) and age (β = 0.145, *p* = 0.020) independently predicted post-treatment pain (R^2^ = 0.129). Conclusion: A reduction in pain intensity was observed after five-week combined therapy protocol. Due to the absence of a control group and the simultaneous use of multiple modalities, no causal conclusions can be drawn, nor can improvements be attributed to SDT alone. Randomized controlled trials with functional outcomes and long-term follow-up are warranted.

## 1. Introduction

Low back pain and sciatica caused by lumbar disc herniation are common conditions that substantially affect quality of life and daily functioning. Sciatica typically presents as radiating neuropathic pain along the sciatic nerve pathway, beginning in the lower back and extending through the buttock and down the leg. Lumbar disc herniation involves displacement of nucleus pulposus or annulus fibrosus material beyond the intervertebral space, which may compress nerve roots and lead to pain, numbness, or weakness [1,2].

Non-invasive interventions, including various forms of lumbar traction, have long been used in conservative management of radicular pain [3]. Modern decompression systems apply computer-controlled axial distraction, which may reduce intradiscal pressure and increase intervertebral spacing [4]. Spinal decompression therapy (SDT) is one such modality. Physiological models and imaging studies suggest potential biomechanical effects—such as increased disc height, reduced lumbar lordosis, or altered vertebral alignment [5,6,7]—but these mechanisms remain partly hypothetical and inconsistently supported by clinical evidence.

Clinical findings regarding decompression-based interventions are heterogeneous. Some studies report short-term symptom reduction or decreased herniation size in acute cases [8,9], whereas others find no meaningful differences between high-force and low-force traction or between conservative and surgical treatments [2,10]. Surveys indicate that traction-based modalities remain widely used in physiotherapy practice despite this inconsistency [11,12]. Chronic low back pain, defined as pain persisting for ≥12 weeks, presents additional challenges [13], and evidence for decompression-based interventions in chronic radiculopathy is particularly limited. CLBP affects 7–10% of people worldwide, and lumbar radiculopathy represents 5–10% of these cases. In European populations, including Croatia, the lifetime prevalence of sciatica caused by lumbar disc herniation is estimated at 3–5% [14].

Although decompression-based interventions are widely used [15], existing studies vary substantially in design quality, patient populations, and comparator treatments, resulting in inconsistent findings that limit firm conclusions. Some trials demonstrate short-term improvements or imaging-based changes [8,9], whereas others show no advantage over sham traction or standard conservative care [10]. This heterogeneity underscores the need for cautious interpretation and highlights gaps in evidence, especially for chronic lumbar radiculopathy. Understanding outcomes following multimodal protocols may help inform clinical decision-making, although such designs cannot establish causality. The present study aimed to evaluate observed changes in pain intensity following a combined therapy protocol including infrared therapy, high-force SDT, and interferential therapy in adults with CLBP and sciatica due to disc herniation at L4–L5 and/or L5–S1.

Three exploratory hypotheses were examined:

(1) Pain intensity would decrease after the multimodal protocol;
(2) Pain reduction might differ between male and female participants, given inconsistent findings on gender-related pain reporting [16,17];
(3) Younger patients might experience greater reductions, as suggested in some conservative spine-care studies [18].

Due to the study design, these hypotheses cannot be interpreted causally.

This study contributes new descriptive evidence on short-term outcomes following a multimodal therapy protocol in a chronic lumbar radiculopathy population—a group for which existing research is scarce and predominantly focused on acute presentations or unimodal interventions. By reporting real-world pre–post data from a large clinical sample, the study addresses a notable gap in the literature and provides a foundation for future controlled trials.

## 2. Materials and Methods

### 2.1. Study Design

A non-randomized pre–post observational design was used to evaluate treatment outcomes in routine clinical practice. The study was conducted at a single outpatient Spinal Rehabilitation Polyclinic between 19 October 2021 and 19 October 2024. Ethical approval was obtained from the institutional Bioethical Committee, and all participants provided written informed consent prior to enrollment. A control group was not included because all participants had persistent symptoms despite completing standard physiotherapy, and withholding further active treatment was considered clinically inappropriate in this real-world setting. The study was designed to document outcomes of a routinely used multimodal protocol rather than to test efficacy under experimental conditions. Usual-care or waitlist controls were not feasible because patients were referred specifically for active intervention after unsuccessful conservative management, and delaying treatment would have conflicted with routine clinical practice and patient expectations. As a result, the study was intentionally structured as a descriptive pre–post observational analysis, aimed at capturing real-world outcomes rather than establishing causality.

### 2.2. Participants

A total of 234 adults with CLBP back pain and sciatica caused by MRI-confirmed disc herniation at L4–L5 and/or L5–S1 were included. Eligibility criteria required symptoms lasting ≥12 weeks and failure to improve after standard physiotherapy, which included electrotherapy, ultrasound, therapeutic exercise, low-force traction, or analgesic medications. Exclusion criteria were pregnancy, malignancy with bone metastasis, and prior spinal surgery. All participants provided written informed consent.

### 2.3. Procedure

Participants completed ten treatment sessions delivered twice weekly over five weeks.

#### 2.3.1. Therapeutic Protocol

Infrared therapy (10 min)

Applied to the lumbar region to promote local warming and relaxation of paraspinal musculature.

#### 2.3.2. High-Force Spinal Decompression Therapy (15 min) (Figure 1)

Supine position with hips flexed to ~65°.

Upper fixation beneath the costal margin; lower fixation around the pelvic girdle at ASIS level.

Intermittent cycles: 8 s traction/8 s relaxation.

Relaxation force: 5 kg.

Initial traction force: 40–50 kg (~60% body weight).

Increment: ~5 kg per session, depending on tolerance.

Maximum force: 90 kg.

Treatment was continuously monitored and remained painless.

**Figure 1:**
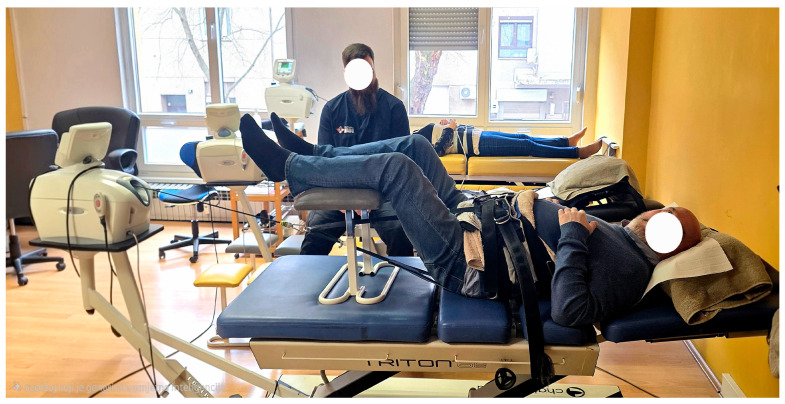
Real life picture of patient in SDT part of procedure.

All applied traction forces in this study (40–90 kg) were selected to remain well within established biomechanical safety limits. In vitro studies demonstrate that the lumbar spine tolerates flexion–distraction loads of approximately 2.8 kN (≈285 kg) before posterior ligamentous and disc structures fail [19], while compressive failure loads range from 0.6 to 15.6 kN (≈60–1590 kg), with mean strengths around 4.8 kN (≈490 kg) [20]. Tensile testing of the annulus fibrosus and ligamentum flavum similarly shows ultimate strengths far exceeding the forces used in therapeutic traction [21,22,23]. Because these data originate from cadaveric specimens—which lack hydration, perfusion, neuromuscular stabilization, and viscoelastic behaviour—they represent conservative lower-bound estimates of in vivo structural tolerance. Furthermore, high-force lumbar traction in the range of 40–60% body weight has been used routinely and safely in clinical practice, with no reports of structural injury when applied under supervision. Accordingly, the forces used in this study operate well below known biomechanical failure thresholds and within accepted clinical safety margins. No adverse events or treatment-related complications were observed during the study.

#### 2.3.3. Interferential Therapy (25 min)

Medium-frequency intersecting currents applied to the lumbar region and lower extremities.

### 2.4. Outcome Measure

Pain intensity was assessed using a verbally administered 0–10 Visual Analogue Scale (VAS) [24] before the first session (baseline VAS; BVAS) and immediately prior to the tenth session (post-treatment VAS; PTVAS). No functional, disability, or quality of life outcomes were collected, limiting interpretation.

### 2.5. Statistical Analysis

Descriptive statistics (mean, median, SD, range) were calculated for age, baseline VAS (BVAS), post-treatment VAS (PTVAS), and VAS difference (DVAS), stratified by gender. Normality was assessed using the Shapiro–Wilk test. Due to non-normal distributions, the Mann-Whitney U test was used to examine gender differences in age and VAS variables, while the chi-square test assessed the association between gender and the side of lumbosacral symptoms.

The Wilcoxon signed-rank test was used to compare baseline VAS (BVAS) and post-therapy VAS (PTVAS), with effect sizes reported as rank biserial correlations. To explore predictors of post-treatment pain, a linear regression model was fitted with PTVAS as the dependent variable and age, gender, duration of symptom, and BVAS as predictors. Assumptions for linear regression were checked and met, including normality of residuals (Shapiro-Wilk and Q-Q plot), independence (Durbin-Watson test), multicollinearity (VIF), homoscedasticity (residual plots), and outlier influence (Cook’s distance). Statistical analysis was conducted using Jamovi (version 2.3.28, an open-source statistical software (The jamovi project, 2025, https://www.jamovi.org)), with statistical significance set at α = 0.05.

## 3. Results

### 3.1. Participant Characteristics

**Figure 2:**
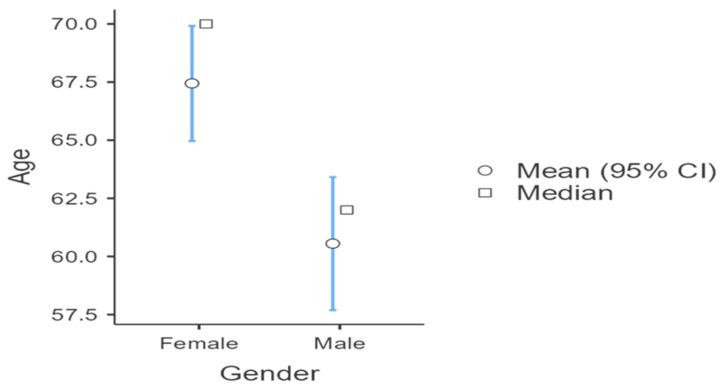
Age distribution by gender (mean with 95% CI and median).

A total of 234 participants with CLBP and sciatica due to disc herniation at L4–L5 or L5–S1 were included, comprising 122 females (52.1%) and 112 males (47.9%) (Table 1). Female participants were older than males (Figure 2) (M = 67.44, SD = 13.94 vs. M = 60.55, SD = 15.44, U = 5055, *p* < 0.001, r = −0.26). Baseline pain intensity, measured by the Visual Analogue Scale (VAS), was high in both sexes (females: M = 7.65, SD = 1.61; males: M = 7.41, SD = 1.62) and did not differ significantly (U = 6266, *p* = 0.266). Post-therapy VAS scores were comparable between sexes (females: M = 5.42, SD = 1.60; males: M = 5.36, SD = 1.26; U = 6597, *p* = 0.640), as was pain reduction (VAS difference: females M = 2.23, SD = 1.94; males M = 2.05, SD = 1.59; U = 6711, *p* = 0.813).

### 3.2. Symptom Laterality

Left-sided lumboischialgia was more common (59.4%) than right-sided (40.6%). Distribution by sex was comparable: females 57.4% left-sided, males 61.6% left-sided (χ^2^(1) = 0.43, *p* = 0.51) (Table 2).

Mann–Whitney U tests (Table 3) demonstrated a statistically significant difference in age between genders (U = 5055, *p* < 0.001), with females being older than males and a small-to-moderate effect size (rank biserial correlation = −0.26). In contrast, no statistically significant gender differences were observed for baseline pain intensity (baseline VAS; U = 6266, *p* = 0.266), post-therapy pain intensity (post-therapy VAS; U = 6597, *p* = 0.640), or pain reduction (VAS difference; U = 6711, *p* = 0.813).

### 3.3. Pain Reduction After Therapy

**Figure 3:**
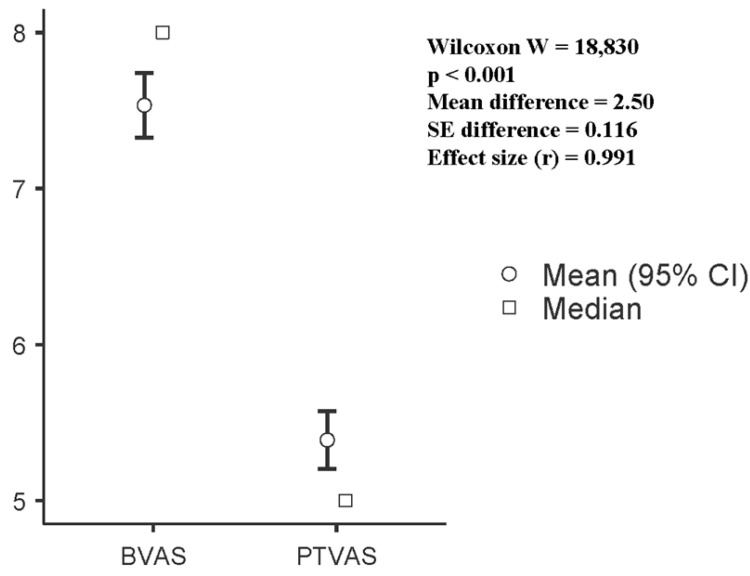
Results of Wilcoxon rank test between baseline VAS and post-therapy VAS.

Pain intensity decreased significantly after nine sessions of combined decompression therapy. The Wilcoxon signed rank test indicated a robust reduction in VAS scores (W = 18,830, *p* < 0.001) (Figure 3). The unweighted mean reduction in pain was 2.5 points, while the weighted mean reduction was 2.14 points. The difference between these estimates reflects the weighting procedure, which assigns greater influence to participants with more complete or stable data; in this sample, those participants tended to show slightly smaller improvements, lowering the weighted estimate; it reflects the rank based weighting inherent to the Wilcoxon test and is not a clinical measure. Overall, the findings demonstrate a clinically meaningful reduction in pain across the cohort, independent of gender or symptom laterality. These results indicate that combined decompression therapy produced a clinically meaningful improvement in pain across the cohort, independent of sex or symptom side.

Figure 2 further illustrates the distribution of age by gender, showing both mean values with 95% confidence intervals and medians. Female participants were older on average than male participants, with higher mean and median ages. The limited overlap of the confidence intervals visually supports the statistically significant gender difference in age reported in Table 3, indicating that females in the sample were significantly older than males.

A linear regression model was fitted to examine predictors of post-therapy pain intensity (PTVAS), with gender, age, duration of symptoms, and baseline VAS (BVAS) entered as independent variables. The model was statistically significant, F(4, 229) = 8.61, *p* < 0.001, accounting for 13.1% of the variance in PTVAS (R^2^ = 0.131; adjusted R^2^ = 0.115).

Baseline pain intensity was the strongest predictor (B = 0.280, *p* < 0.001, β = 0.312), indicating that participants with higher initial pain tended to report higher pain levels after therapy. Age also contributed significantly (B = 0.015, *p* = 0.019, β = 0.152), suggesting that older individuals experienced slightly higher post-treatment pain after controlling for other variables. In contrast, gender (*p* = 0.567) and duration of symptoms (*p* = 0.830) were not significant predictors of PTVAS.

Full regression results are presented in Table 4.

## 4. Discussion

The aim of this study was to evaluate short-term changes in pain intensity following a multimodal rehabilitation protocol incorporating infrared therapy, high-force spinal decompression therapy (SDT), and interferential therapy in adults with CLBP and sciatica due to lumbar disc herniation at L4–L5 and/or L5–S1. The main finding was a statistically significant and clinically meaningful reduction in pain intensity, with an average VAS decrease of 2.5 points, exceeding the minimal clinically important change (MCIC) of 2.0 points for CLBP [13].

When compared with the existing literature, the present findings are broadly consistent with studies reporting short-term symptomatic improvement following traction-based or multimodal physiotherapy interventions. Previous research has demonstrated reductions in pain intensity or herniation size in selected patients undergoing decompression-based treatments [7,8,17], although results across studies remain heterogeneous due to differences in methodology, patient selection, and treatment parameters. Surveys of physiotherapists in the United States and United Kingdom indicate that traction-based modalities remain widely used in clinical practice despite inconsistent evidence [10,11]. The current study contributes to this body of work by examining a high-force, machine-controlled protocol in a chronic population that had not improved with standard physiotherapy, providing real-world data from a clinically relevant subgroup.

Interpretation of underlying mechanisms must remain cautious. Because the present study did not include physiological measurements, biomechanical assessments, or imaging follow-up, mechanistic explanations cannot be inferred from the results. Although previous imaging and experimental studies have reported changes in disc height, foraminal dimensions, lumbar alignment [5,6], intradiscal pressure [16], or nutrient transport within degenerated discs [4], the current design does not allow for conclusions regarding structural or physiological effects.

Pain reduction was observed across demographic groups. No significant differences were found between men and women in baseline pain, post-treatment pain, or magnitude of improvement, consistent with previous findings suggesting that gender does not substantially influence short-term response to traction-based interventions [10,11]. Age showed a small but statistically significant association with post-treatment pain, indicating that older individuals may experience slightly less improvement; however, the effect size was modest, and the intervention remained beneficial across all age groups.

Several limitations must be acknowledged. First, the intervention was multimodal, combining infrared therapy, high-force SDT, and interferential therapy, preventing attribution of the observed effects to SDT alone. Second, the absence of a control group limits causal inference and makes it impossible to distinguish treatment effects from natural symptom fluctuation, regression to the mean, placebo responses, or nonspecific therapeutic influences. Third, the exclusive use of VAS as the sole outcome measure is a major limitation. CLBP affects multiple domains—including disability, mobility, and quality of life—that were not assessed. The absence of validated functional instruments such as the Oswestry Disability Index (ODI) or Roland–Morris Disability Questionnaire (RMDQ) restricts interpretation to pain intensity alone. Fourth, no imaging follow-up was performed, limiting insight into potential structural changes that have been documented in previous decompression studies [5,6,7,8,9,16,17]. Fifth, the regression model included only a limited set of predictors, leaving many potentially relevant clinical, psychosocial, and biomechanical variables unexamined [16,17,18]. Finally, the study was conducted in a single clinical centre, which may limit generalizability.

Despite these limitations, the findings provide preliminary real-world descriptive evidence that a supervised, high-force decompression-based multimodal protocol may contribute to short-term pain reduction in patients with chronic lumbar radiculopathy who have not improved with standard physiotherapy. Future controlled studies incorporating functional and quality of life outcomes, imaging assessments, and long-term follow-up are needed to clarify the specific contribution of SDT and determine the clinical relevance and durability of the observed improvements.

## 5. Conclusions

This study evaluated short-term changes in pain intensity following a multimodal rehabilitation protocol combining infrared therapy, high-force spinal decompression therapy (SDT), and interferential therapy in adults with CLBP and sciatica due to lumbar disc herniation. Participants experienced statistically significant and clinically meaningful reductions in pain intensity, despite having persistent symptoms after standard physiotherapy. These findings suggest that a supervised multimodal approach may be associated with short-term symptomatic relief in a chronic population that is often difficult to treat.

However, the results must be interpreted within the constraints of the study design. Because all therapeutic modalities were applied simultaneously, the specific contribution of SDT cannot be determined. The absence of a control group further limits causal inference, and improvements may reflect natural symptom fluctuation, regression to the mean, placebo effects, or nonspecific therapeutic influences. The exclusive use of VAS restricts interpretation to pain intensity alone, without insight into disability, functional capacity, or quality of life. The lack of imaging follow-up and long-term monitoring also prevents conclusions regarding structural changes or durability of effects.

Potential bias related to the clinical setting was minimized through several measures. Although one of the authors owns the clinic where the intervention was conducted, data collection, data entry, and statistical analysis were performed by team members who were not involved in treatment delivery or clinical decision-making. Treating clinicians did not participate in outcome assessment, and all analyses followed predefined procedures. These steps were implemented to ensure independence of data handling and reduce the risk of systematic bias.

Despite these limitations, the study provides real-world descriptive evidence that a multimodal decompression-based protocol may offer short-term pain reduction in individuals with chronic lumbar radiculopathy who have not responded to standard conservative care.

Future research should employ randomized controlled designs, incorporate validated functional and quality of life measures, include imaging assessments, and evaluate long-term outcomes to clarify the specific role and clinical relevance of high-force SDT within comprehensive rehabilitation programs.

## Figures and Tables

**Table 1:** Descriptive sample parameters of numerical variables.

Variable	Gender	*n*	Mean	Median	SD	Min	Max
**Age (years)**	Female	122	67.44	70.00	13.94	20.00	97.00
	Male	112	60.55	62.00	15.44	24.00	90.00
**Baseline VAS**	Female	122	7.65	8.00	1.61	3.00	10.00
	Male	112	7.41	8.00	1.62	4.00	10.00
**Post-therapy VAS**	Female	122	5.42	5.00	1.60	1.00	10.00
	Male	112	5.36	5.00	1.26	2.00	9.00
**VAS difference**	Female	122	2.23	2.00	1.94	−1.00	9.00
	Male	112	2.05	2.00	1.59	−1.00	8.00

**Table 2:** Distribution of lumboischialgia side by gender.

Gender	Left, *n* (%)	Right, *n* (%)	Total
Female	70 (57.4%)	52 (42.6%)	122
Male	69 (61.6%)	43 (38.4%)	112
Total	139 (59.4%)	95 (40.6%)	234

**Table 3:** Results of Mann-Whitney U tests for age and VAS variables by gender.

Variable	Test Statistic (U)	*p*-Value	Effect Size (Rank Biserial r)
Age	5055	<0.001	−0.2602
Baseline VAS	6266	0.266	−0.0828
Post-therapy VAS	6597	0.640	−0.0344
VAS difference	6711	0.813	−0.0177

**Table 4:** Linear regression model predicting post-therapy pain (PTVAS).

Model Fit: F(4, 229) = 8.61, *p* < 0.001; R = 0.361; R^2^ = 0.131; R^2^adj = 0.115					
Predictor	B	SE	t	*p*-Value	β
Gender (Male vs. Female)	0.105	0.183	0.573	0.567	0.073
Age	0.015	0.006	2.371	0.019	0.152
Duration of symptoms	0.00001	0.00005	0.215	0.830	0.014
BVAS	0.280	0.056	4.987	<0.001	0.312

## Data Availability

Raw data can be requested from the corresponding author.

## Abbreviations

The following abbreviations are used in this manuscript:

Spinal decompression therapy	SDT
Chronic low back pain	CLBP
